# Health literacy of Sesotho-speaking patients diagnosed with chronic conditions in South Africa

**DOI:** 10.4102/phcfm.v14i1.3627

**Published:** 2022-12-20

**Authors:** Mita S. Mofokeng, Marianne Reid, Melanie Pienaar, Mariette Nel

**Affiliations:** 1 School of Nursing, Faculty of Health Science, University of Free State, Bloemfontein, South Africa; 2 School of Biostaticians, Faculty of Health Science, University of Free State, Bloemfontein, South Africa

**Keywords:** health literacy, public healthcare facilities, SHLT, chronic conditions, Sesotho speaker, healthcare provider, adherence

## Abstract

**Background:**

Health literacy influences patients’ health outcomes, as their ability to read, interpret and apply health information associated with health-related decision-making. These decision-making skills need to be made up by patients diagnosed with chronic conditions – also Sesotho-speaking patients receiving treatment in public primary health care environments.

**Aim:**

The study aimed to assess the health literacy of Sesotho-speaking patients diagnosed with chronic conditions and to establish the associations between the sociodemographic data of patients and items of a health literacy test.

**Setting:**

This study was conducted in public healthcare (PHC) facilities in the Free State province, South Africa.

**Methodology:**

A quantitative descriptive cross-sectional design involved conveniently sampled patients with chronic conditions (*n* = 264) who were being treated at PHC facilities (*n* = 12) in the Setsoto subdistrict and who completed the Sesotho Health Literacy test during a structured interview. Descriptive statistics were calculated per group and compared by means of chi-square or Fisher’s exact test and Kruskal–Wallis test.

**Results:**

Test results indicate high literacy levels in 35.6% (*n* = 94), moderate health literacy levels in 43.6% (*n* = 115) and low health literacy levels in 20.8% (*n* = 55) of participants. No association (*p* = 0.14) was found between health literacy level and gender or chronic conditions or between health literacy level and the participants’ inability to read due to poor eyesight (*p* = 0.21). Positive associations (*p* ≤ 0.01) were established between a health literacy level and age and between health literacy level and education: participants with a South African School Grade Level 9–12 (*p* ≤ 0.01) had higher health literacy levels.

**Conclusion:**

Healthcare providers caring for Sesotho-speaking patients need to be sensitive about their patients’ health literacy levels, as it may play a role in their health outcomes.

**Contribution:**

The value of the findings reported lies in the possibility of rapidly appraising the health literacy levels of a large indigenous population in South Africa diagnosed with chronic conditions.

## Introduction

Like many other low- and middle-income countries, South Africa has high mortality and hospitalisation rates related to chronic conditions,^1,2^ which have led the country to revise funds allocated to the public healthcare (PHC) sector to combat the increasing burden of disease.^2^ The increasing number of patients diagnosed with chronic conditions has caused severe pressure on the healthcare system,^2^ and it is estimated that, in the Free State province, South Africa, alone, 24% of its population has been diagnosed with chronic conditions.^1^

It is estimated that 72% of the population in the Free State are Sesotho speakers, who are predominantly black South Africans.^3^ Sesotho is an African language that originated as early as the 1300s and corresponds with the four significant ethnic divisions among black South Africans.^4^ Sesotho is one of the 11 official languages spoken in South Africa^5^ and one of the first black languages in South Africa to be presented in a written format,^6^ which accentuates the significance of knowing this population’s health literacy, especially given the high percentage of this in a population diagnosed with chronic conditions.

Health literacy in South Africa is a relatively new concept, on which limited research has been done, especially in the PHC setting.^7^ The definition of health literacy endorsed by the World Health Organization refers to the:

[*P*]ersonal characteristics and social resources needed for individuals and communities to access, understand, appraise and use information and services to make decisions about health. Health literacy includes the capacity to communicate, assert and enact these decisions.^8^ (p. 12)

Patients diagnosed with chronic conditions are often expected to read and interpret treatment instructions^7,9^ and make informed health decisions.^10^ Health literacy does not only begin and end with patients having the ability to read and interpret treatment instructions but also how to navigate the healthcare system^11^ and read educational and food labels to make well-informed decisions regarding their health. Patients diagnosed with chronic conditions are also expected to modify their lifestyle according to their diagnosis by eating healthy and engaging in activities that will benefit their health outcomes.^12^ Literature indicates that inadequate health literacy may impact treatment adherence and self-management and decision-making abilities and has been linked to frequent hospitalisation^13^ and poor health outcomes.^14,15,16^ In the South African context, patients diagnosed with chronic conditions mostly receive care and treatment at public facilities of their choice, where they consult healthcare providers.^17^

Healthcare providers in a PHC setting are expected to manage and treat patients according to their chronic conditions by prescribing medication and providing effective health education to improve patients’ health outcomes.^17^ The high number of patients (84%) currently diagnosed with chronic conditions and seeking care and treatment at PHC facilities^9^ has risen further since 2012, when PHC reengineering was implemented, and a focus on providing PHC in PHC facilities was renewed.^18,19^ The number of patient visits to PHCs in the country increased from 68 million in 1998 to 120 million in 2015,^20^ which has contributed to a high patient load that makes it difficult for healthcare providers to provide quality care.

Taking patients’ health literacy levels into consideration may improve healthcare quality. Fortunately, a validated Sesotho Health Literacy Test (SHLT) is available to measure the general health literacy of all Sesotho-speaking patients. The SHLT was developed using a mixed-methods design. The theoretical framework guiding the development presented the opportunity for health literacy test items to identify specific competencies and skills required within the South African public health care system.^21^ Validity testing of the SHLT focussed on the understandability of SHLT items, item response, factor analysis and convergent and predictive validity of test items. Validation results indicated or endorsed the usability of the SHLT.^22^

This study aimed to assess the health literacy of Sesotho-speaking patients diagnosed with chronic conditions and to determine whether there are associations between the sociodemographic data of patients and items on the SHLT in Setsoto, Free State province, South Africa.

## Research methods and design

### Study design

A descriptive cross-sectional design was applied in this study, which was conducted over two weeks in August 2019.

### Setting

The population consisted of patients diagnosed with a chronic condition attending 12 PHC facilities in Setsoto, which is a subdistrict of the Thabo Mofutsanyana district of the Free State province of South Africa.

### Study population and sampling strategy

All the subdistrict PHC facilities (*N* = 12) are visited by an estimated 6390 patients diagnosed with chronic conditions monthly. Patients at all 12 PHC facilities were proportionally sampled, and the participants were conveniently sampled from each facility, as indicated in Table 1.^23^ It is problematic to do random sampling in a clinic setup. When an interview was complete, the next participant was approached for participation. This process continued throughout the day during clinic hours. Because of logistical constraints such as time and budget, the researcher could only sample 300 participants. Patients were recruited in the waiting area of each PHC facility by the first author and fieldworkers while the patients waited for a routine follow-up appointment. If patients opted to take part in the study, they were taken to a private area to maintain confidentiality; written informed consent was obtained if they met the following inclusion criteria: participants aged at least 18 years and had been diagnosed with a chronic condition, such as hypertension, asthma, diabetes, arthritis, epilepsy, peptic ulcers, human immunodeficiency virus (HIV) or heart (congestive heart failure) and mental conditions (depression, bipolar disorder, schizophrenia). Finally, participants also had to be Sesotho first-language speakers to be included in the study.

**TABLE 1:** Distribution of proportionally sampled participants per public healthcare facility, as well as conveniently sampled participants at a public healthcare facility. ^23^

PHF in Setsoto subdistrict	Estimated population attendance per month	Proportional sample	Convenient sample
PHF 1	578	27	25
PHF 2	617	29	29
PHF 3	61	3	3
PHF 4	519	24	23
PHF 5	556	26	26
PHF 6	715	34	34
PHF 7	377	18	18
PHF 8	97	5	5
PHF 9	237	11	11
PHF 10	836	39	38
PHF 11	1054	49	25
PHF 12	743	35	27

**Total**	**6390**	**300**	**264** †

*Source:* Mofokeng MS. Health literacy of Sesotho-speaking patients diagnosed with chronic conditions: Setsoto, Free State province [homepage on the internet]. Bloemfontein: University of the Free State; 2020 [cited 2022 Aug 22]. Available from: https://scholar.ufs.ac.za/xmlui/handle/11660/11039

† , Challenges faced in PHC facilities prevented the researcher from strictly applying the advised proportional size of the sample and/or facility.

PHF, public health care facilities.

### Data collection

**FIGURE 1:**
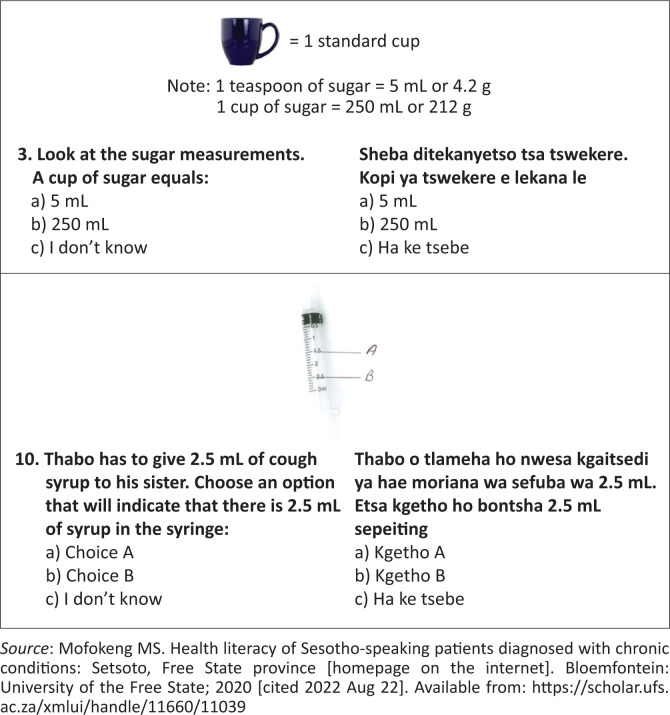
Example of English-translated questions in Sesotho Health Literacy Test.^23^

Data were collected on site in private areas at all PHC facilities (*N* = 12) from 264 participants by the first author and two trained fieldworkers, who were also Sesotho first-language speakers. Data were collected by means of the SHLT. The SHLT is a pre-existing and validated tool developed for Sesotho home-language speakers. The SHLT determines the general health literacy of Sesotho-speaking patients.^21,22^ The test comprises two sections: Section A gathers demographic data about participants, which include gender, age and the highest academic level attained. Participants also had to indicate whether they struggled to read because of poor eyesight and report chronic condition(s) they had been diagnosed with. Section B of the SHLT comprised 10 multiple choice questions – participants had to select the correct answer from three possible answers. The first author and Sesotho-speaking fieldworkers guided participants individually to complete the SHLT. A few examples of the questions are presented in Figure 1.^23^ Before the main study, the test was piloted, with the pilot serving as an additional training platform for the fieldworkers to conduct the test. Data collected (*n* = 12) during the pilot study were not included in the study data.

Questions 1–6 of the SHLT measured appraisal of health information, while questions 7–10 measured understanding of health information. The full version of the SHLT Sesotho or English may be requested from the corresponding author. The 10 multiple choice questions had pre-determined correct answers. The scores were interpreted as follows: A score of < 6: Low health literacy; 6–8: Moderate health literacy and > 8: High health literacy.^21^

### Data analysis

The authors limited measurement errors by confirming captured data twice on an Excel spreadsheet. Data were analysed by the Department of Biostatistics at the [University of The Free State] using descriptive statistics, namely frequencies and percentages for categorical and numerical data results. The article will report summary statistics, median and interquartile range (IQR). The groups were compared by means of chi-square test or Fisher’s exact test for categorical data and Kruskal–Wallis test for numerical data. Data analysis of this study was done with SAS software (copyright, SAS Institute Inc. SAS and all other SAS Institute Inc. products or service names are registered trademarks or trademarks of SAS Institute Inc., Cary NC, USA).

### Ethical considerations

Ethical approval for the study was granted by the University of the Free State Health Sciences Research Ethics Committee (UFS-HSD2019/0478/3007), and permission to conduct the study was obtained from the Free State Department of Health. Information leaflets were handed out to all patients to explain the study, and informed consent was signed by all participants before they took part in the study. The participants were given the option to withdraw from the study at any time, and they were assured that they would not suffer any penalties if they did so. Each participant was assigned a number, thereby ensuring that their identity and information would be kept private.

## Results

### Demographic characteristics

Table 2^23^ depicts the demographic characteristics of participants. Women accounted for the majority (82.6%) of participants in the study. The ages of the participants ranged between 20 and 93 years, with a median age of 43 years. The highest academic levels achieved by participants were segregated according to the highest school grade level passed. The majority (56.8%; *n* = 150) indicated having passed Grade 9–12. To exclude poor eyesight as a variable that could influence health literacy, it was determined that 46.6% (*n* = 123) of participants in this study reported problems with eyesight. The participants had been diagnosed with a wide range of chronic conditions, with HIV being the most prevalent (61%; *n* = 164).

**TABLE 2:** Demographic characteristics (*N* = 264). ^23^

Variable	Value			
	*n*	%	median	IQR
Female	218	82.6	-	-
Age	-	-	43	32–56
Highest school grade level passed	-	-	9	7–11
Struggles to read because of poor eyesight	123	46.6	-	-
**Chronic conditions**				
Hypertension	99	37.5	-	-
HIV	164	62.1	-	-
Asthma	10	3.8	-	-
Diabetes	24	9.1	-	-
Arthritis	13	4.9	-	-
Mental health condition	4	1.5	-	-
Epilepsy	9	3.4	-	-
Peptic ulcer	2	0.8	-	-
Heart condition	2	0.8	-	-

*Source:* Mofokeng MS. Health literacy of Sesotho-speaking patients diagnosed with chronic conditions: Setsoto, Free State province [homepage on the internet]. Bloemfontein: University of the Free State; 2020 [cited 2022 Aug 22]. Available from: https://scholar.ufs.ac.za/xmlui/handle/11660/11039

IQR, interqaurtile range; HIV, human immunodeficiency virus.

* , *p*-value is statistically significant.

### Sesotho Health Literacy Test results

**FIGURE 2:**
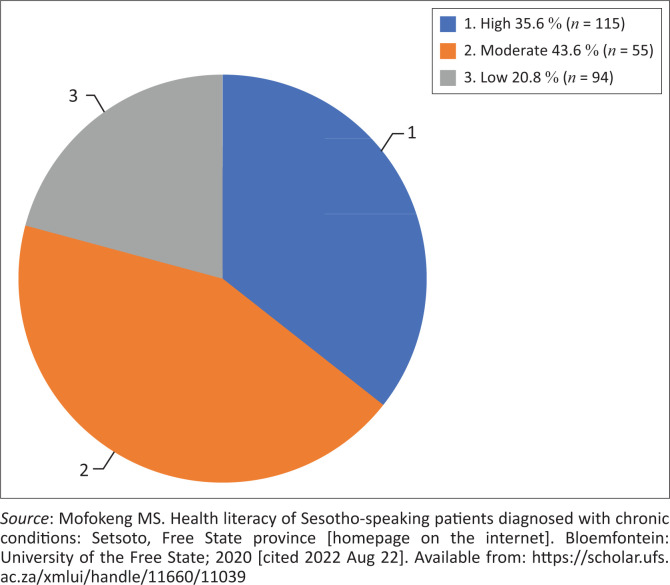
Sesotho Health Literacy Test scores (*N* = 264). ^23^

The data presented in Figure 2^23^ illustrate the literacy levels of participants according to their responses to items of the SHLT: A score of < 6: Low health literacy; 6–8: Moderate health literacy and > 8: High health literacy.

The finding is that most (43.6%; *n* = 115) of the participants in the study could be classified as possessing moderate health literacy, with just more than a third (35.6%; *n* = 94) being classified with high health literacy. This finding suggests that a significant portion (43.6%) of participants may need assistance with conceptualising health literacy-related information, and a further 21%, who could be classified as having low health literacy, may need structured guidance to interpret health-related information.

### Association between health literacy classification and demographic characteristics

The results in Table 3^23^ suggest that a significant association (*p* < 0.05)* was established between health literacy and age, highest academic level obtained and items assessing appraisal and understanding. No significant difference was found regarding the chronic conditions and the level of health literacy.

**TABLE 3:** Association between health literacy and demographic data as well as health literacy and specific chronic conditions. ^23^

Association	Low health literacy				Moderate health literacy				High health literacy				*P*-value
	*n*	%	median	IQR	*n*	%	median	IQR	*n*	%	median	IQR	
**Age**	-	-	56	43–60	-	-	43	32–56	-	-	55	30–46	< 0.01*
**Gender**	-	-	-	-	-	-	-	-	-	-	-	-	0.14
Female	14	25.4	-	-	20	17.3	-	-	12	12.7	-	-	-
Male	41	74.5	-	-	95	82.6	-	-	82	87.2	-	-	-
**Highest school grade level passed**	-	-	7	2–9	-	-	9	6–11	-	-	11	9–12	< 0.01*
**Appraisal items**	-	-	3	2–3	-	-	4	3–4	-	-	5	4–5	< 0.01*
**Understanding items**	-	-	2	1–2	-	-	3	3–3	-	-	4	3–4	< 0.01*
**Chronic conditions**													
Hypertension	25	45.5	-	-	42	36.5	-	-	32	34.0	-	-	0.37
HIV	28	50.9	-	-	79	68.7	-	-	57	60.64	-	-	0.08
Asthma	3	5.5	-	-	4	3.5	-	-	3	3.2	-	-	0.77
Diabetes	5	9.1	-	-	9	7.8	-	-	10	10.6	-	-	0.78
Arthritis	3	5.5	-	-	7	6.1	-	-	3	3.2	-	-	0.67
Mental condition (depression, bipolar disorder, schizophrenia)	1	1.8	-	-	3	2.6	-	-	0	0.0	-	-	0.27
Epilepsy	3	5.5	-	-	5	4.4	-	-	1	1.1	-	-	0.26
Peptic ulcer	1	1.8	-	-	0	0.0	-	-	1	1.1	-	-	0.32
Heart condition (congestive heart failure)	0	0.0	-	-	1	0.9	-	-	1	1.1	-	-	1.00

*Source:* Mofokeng MS. Health literacy of Sesotho-speaking patients diagnosed with chronic conditions: Setsoto, Free State province [homepage on the internet]. Bloemfontein: University of the Free State; 2020 [cited 2022 Aug 22]. Available from: https://scholar.ufs.ac.za/xmlui/handle/11660/11039

IQR, interquartile range; HIV, human immunodeficiency virus.

* , *p*-value is statistically significant.

## Discussion

The study aimed to assess the health literacy of Sesotho speakers who had been diagnosed with chronic conditions at PHC facilities. The SHLT, which was used for this assessment, is culturally and language appropriate for this specific indigenous group in South Africa.

A notable percentage (43.6%) of the participants were rated as having moderate health literacy (6–8) and 20.8% were rated as having low health literacy (<6). A high probability exists that patients may experience challenges with understanding and applying the health information they receive and navigating the healthcare system. In a South African study where hypertension health literacy was measured, the results showed that some participants (19%) had a challenge with reading and writing which had a negative impact on following and understanding treatment instructions.^12^ Another study conducted in the Eastern Cape, South Africa, also presented similar findings where the majority of participants (71.4%) were rated with moderate health literacy stressing the challenges participants have with reading and interpreting treatment instructions.^9^

A study conducted among indigenous Australian people living with chronic conditions found that younger people – aged no more than 55 years – had higher health literacy levels.^24^ In the Australian study, this finding was ascribed to exposure to educational opportunities. The current study did not explore associations with educational opportunities. However, the positive correlation between education and health literacy levels is widely accepted.^9^

This study found no association between health literacy and gender among its Sesotho-speaking participants. An Iranian study conducted with patients diagnosed with Type 2 diabetes also found no notable gender association with health literacy and reported low health literacy for both gender groups.^25^ Furthermore, no association was found between health literacy and chronic conditions within the reported study, whereas in the South African study focussing on health literacy and patients with hypertension, a relatively low percentage (19%) of these patients presented with poor hypertension health literacy levels.^12^

Poor eyesight is often associated with chronic conditions such as diabetes^26^ and hypertension. Therefore, poor eyesight may impact patients negatively, specifically when they are expected to follow written instructions.^27,28,29^ The authors of this study purposefully wanted to exclude eyesight as a variable. No association was established between health literacy and the participants’ inability to read because of poor vision.

The findings of this study suggest, furthermore, a positive correlation between health literacy and education levels. A sound educational background creates the foundation for reading and understanding and applying information.^13,30^ Various studies^31,32^ confirm that patients with an education could interpret treatment instructions and apply health information proficiently, thus improving their health outcomes.

Health literacy has been found to impact patients’ health outcomes significantly; patients often feel embarrassed to clarify information provided by healthcare providers.^7^ It is therefore important to identify a patient’s health literacy level and, for healthcare providers, to manage patients accordingly.^9,30,33^ Low health literacy in patients often goes unnoticed by healthcare providers,^34,35^ despite the time healthcare providers spend promoting the health of patients diagnosed with chronic conditions and helping them to navigate the healthcare system more effectively.^31^

### Limitations

The authors acknowledge that the findings reflect the health literacy levels of only Sesotho-speaking patients diagnosed with chronic conditions within a subdistrict in Free State province, South Africa. The result of this study is not representative of the broader Sesotho-speaking population in South Africa. An enlarged sample size may be used within future studies enabling generalisation of results.

## Conclusion

Health literacy influences patients’ health outcomes. Knowing the health literacy level of patients receiving healthcare is an established way of improving the health outcomes of patients diagnosed with chronic conditions. The low health literacy levels of the Sesotho-speaking population reported in this paper may compromise their ability to read, interpret, apply and make health-related decisions leading to adverse health outcomes. Health literacy-related skills are required for patients diagnosed with chronic conditions to optimise health outcomes.

The value of the findings reported lies in the possibility of rapidly appraising the health literacy levels of a large indigenous population in South Africa diagnosed with chronic conditions. By taking into account these patients’ health literacy levels, it would be possible to provide better healthcare to this group of almost 4 million South Africans out of 55 million.^3^ However, doing so would necessitate a renewed sensitivity by healthcare providers regarding the influence of health literacy on health outcomes. A simple request to patients to explain back concepts that have been discussed could potentially assist patients to make more informed health decisions. The health outcomes of patients with chronic conditions can, therefore, be improved when patients’ health literacy levels are known and taken into consideration.
